# A comparative study on various cell sources for constructing tissue-engineered meniscus

**DOI:** 10.3389/fbioe.2023.1128762

**Published:** 2023-03-16

**Authors:** Rui Zheng, Daiying Song, Yangfan Ding, Binbin Sun, Changrui Lu, Xiumei Mo, Hui Xu, Yu Liu, Jinglei Wu

**Affiliations:** ^1^ Department of Dermatology, Shanghai Ninth People’s Hospital, Shanghai Jiao Tong University School of Medicine, Shanghai, China; ^2^ Shanghai Engineering Research Center of Nano-Biomaterials and Regenerative Medicine, Department of Biomedical Engineering, Donghua University, Shanghai, China; ^3^ Research Institute of Plastic Surgery, Wei Fang Medical College, Weifang, China; ^4^ Department of Plastic and Reconstructive Surgery, Shanghai Key Laboratory of Tissue Engineering, Shanghai Ninth People’s Hospital, Shanghai Jiao Tong University School of Medicine, Shanghai, China

**Keywords:** meniscus, scaffold, cell source, tissue engineering, fibrocartilage tissue

## Abstract

Injury to the meniscus is a common occurrence in the knee joint and its management remains a significant challenge in the clinic. Appropriate cell source is essential to cell-based tissue regeneration and cell therapy. Herein, three commonly used cell sources, namely, bone marrow mesenchymal stem cell (BMSC), adipose-derived stem cell (ADSC), and articular chondrocyte, were comparatively evaluated to determine their potential for engineered meniscus tissue in the absence of growth factor stimulus. Cells were seeded on electrospun nanofiber yarn scaffolds that share similar aligned fibrous configurations with native meniscus tissue for constructing meniscus tissue *in vitro*. Our results show that cells proliferated robustly along nanofiber yarns to form organized cell-scaffold constructs, which recapitulate the typical circumferential fiber bundles of native meniscus. Chondrocytes exhibited different proliferative characteristics and formed engineered tissues with distinct biochemical and biomechanical properties compared to BMSC and ADSC. Chondrocytes maintained good chondrogenesis gene expression profiles and produced significantly increased chondrogenic matrix and form mature cartilage-like tissue as revealed by typical cartilage lacunae. In contrast, stem cells underwent predominately fibroblastic differentiation and generated greater collagen, which contributes to improved tensile strengths of cell-scaffold constructs in comparison to the chondrocyte. ADSC showed greater proliferative activity and increased collagen production than BMSC. These findings indicate that chondrocytes are superior to stem cells for constructing chondrogenic tissues while the latter is feasible to form fibroblastic tissue. Combination of chondrocytes and stem cells might be a possible solution to construct fibrocartilage tissue and meniscus repair and regeneration.

## 1 Introduction

The meniscus is crescent fibrocartilaginous tissue that is firmly anchored onto the tibial plateau within the knee joint. It plays a critical role in the preservation of the cartilage and the maintenance of structural stability of the knee joint. However, the meniscus is vulnerable to various sports-related traumatic injuries and degenerative diseases. What is more, it has poor self-healing capacity due to the limited blood supply caused by its largely avascular nature. Damage to the meniscus is highly associated with cartilage degeneration, which finally leads to the development of osteoarthritis (Monibi and Cook, 2017). Repair and regeneration of the meniscus is a significant challenge in clinical practice. Tissue engineering that combines scaffolds, cells, and growth factors represents a promising approach to repair and regenerate various tissues. Recently, meniscus tissue engineering has gained increasing attention because it is not only capable of constructing engineered meniscus tissues *in vitro* and/or *in vivo* for implanting but also provides a feasible platform for investigating meniscus cellular biology and cell-matrix interactions (Bilgen et al., 2018).

Scaffolds are essential in structural-oriented approaches to engineering meniscus tissues because they provide three-dimensional templates for cell growth and tissue formation (Bilgen et al., 2018; Wang J et al., 2022). Structurally, the meniscus is featured by the unique gross crescent shape with hierarchical and intertwined fibrous ECM, which enables its good capability to resist complex loads in the knee joint (Li et al., 2017). Molding biomaterials such as polyurethane (Liu et al., 2012), collagen (Puetzer and Bonassar, 2013), and alginate (Puetzer et al., 2012) in custom-made molds could achieve scaffolds reconstructing the meniscus with crescent shape. Recently, three-dimensional printing technique has also shown advantages in fabricating crescent-shaped scaffolds for meniscus regeneration (Li et al., 2021a; Chae et al., 2021; Deng et al., 2021). Despite good reconstruction of the anatomic shape, these scaffolds are not appropriately recapitulating the fibrous structure of native meniscus ECM. Recently, we and other groups have reported that electrospun scaffolds show promise in meniscus tissue engineering and repair applications due to their biomimetic fibrous structure (Qu et al., 2017; Li et al., 2020; Wang et al., 2021; Xia et al., 2021; Dorthe et al., 2022). Conventional electrospun scaffolds with either random or aligned fiber orientations support good cell proliferation but do not allow cell infiltration owing to their dense fibrous configurations. Mauck group demonstrated that scaffold porosity, rather than scaffold alignment, plays a critical role in construct maturation and tissue integration in an *in vitro* meniscus defect model (Baker et al., 2012; Ionescu and Mauck, 2013). In line with these findings, our recent studies also indicate that electrospun scaffolds composed of aligned nanofiber yarns not only promote cell maturation (Wang et al., 2021) but also show improved tissue remodeling *in vivo* (Li et al., 2021b). Therefore, electrospun nanofiber yarn scaffolds might represent a good candidate for meniscus repair and regeneration.

Appropriate cell source is another prerequisite for constructing meniscus tissue. However, cellular biology of the meniscus is relatively complex, where cells in the outer periphery are fibroblast-like while cells in the inner portion are chondrocyte-like (Sanchez-Adams and Athanasiou, 2009; Wang T et al., 2022). Accordingly, many cells have been efforted for meniscus tissue engineering. Meniscus cells isolated from animals (Wu et al., 2021; Xia et al., 2021) or harvested from surgical debris (Baker et al., 2009) in clinic are the most commonly used cell source for meniscus tissue engineering. Fibroblasts and chondrocytes are frequently used to evaluate the cytocompatibility and functionality of meniscus scaffolds (Wu et al., 2015). Stem cells are another attractive cell source for constructing meniscus tissue *in vitro* and for scaffold-based or scaffold-free cell therapy for meniscus repair in preclinical trials (Yuan et al., 2017; Elkhenany et al., 2021; Bian et al., 2022; Ding G et al., 2022). While these cells are effective for meniscus tissue engineering, some problems exist. Meniscus cells are known for high heterogeneity due to the diversity of cell populations within the native meniscus. Isolated meniscus cells are difficult to be sorted because of the lack of specific markers (Lee et al., 2020). In addition, clinical use of meniscus cells might be limited by donor tissue shortage. Chondrocytes and fibroblasts represent the dichotomy of meniscus cell phenotype and are efficient in investigating the chondrogenesis and fibroblastic of engineered meniscus tissue, they are genetically distinct from resident cells of the native meniscus. As for stem cells, growth factors are often involved to induce chondrogenic differentiation, which might raise safety concerns in clinical practice. The optimal cell source for meniscus tissue engineering is still under debate (Elkhenany et al., 2021; Lee et al., 2021).

In this study, we aim to compare various cell sources combined with a biomimetic scaffold, namely, electrospun yarn scaffold, for constructing engineered meniscus tissue and providing insight for appropriate cell source for meniscus repair. Our previous studies have proven good efficacy of electrospun yarn scaffolds in constructing fibrocartilaginous tissues. Herein, bone marrow mesenchymal stem cells (BMSCs), adipose stem cells (ADSCs), and articular chondrocytes were seeded on electrospun yarn scaffolds in the absence of growth factor for *in vitro* construction of meniscus tissue. We hypothesize that these cells respond differently to the yarn scaffold in the absence of growth factor stimulus and form distinct engineered tissues. Cell-scaffold interactions include the expression of meniscogenic genes, the secretion of fibrocartilaginous ECM, and biomechanics of engineered meniscus tissues were investigated to determine which cell is optimal for tissue engineered meniscus tissues.

## 2 Materials and methods

### 2.1 Materials

Poly (lactide-co-caprolactone) (PLLA-CL, LA:CL = 75:25) was purchased from Jinan Daigang Biomaterial Co., Jinan, China. 1,1,1,3,3,3-hexafluoro-2-propanol (HFIP) was purchased from Da-Rui FineChemical Co. Ltd., Shanghai, China. DNase (D875698) was obtained from Macklin Inc., Shanghai, China. Magnesium chloride (M8266), phenol/chloroform/isoamyl alcohol (77,619) were purchased from Sigma-Aldrich. Papain (P164463) and pepsin (P110928) were obtained from Aladdin Biochemical Technology Co. Ltd., Shanghai, China. Hydrochloride acid (10011018), and 200-proof ethanol (10009218) were supplied by Sinopharm Chemical Reagent Co., Ltd., Shanghai, China.

### 2.2 Scaffold preparation

Nanofiber yarn scaffold was prepared *via* electrospinning following our previous studies (Li et al., 2021c; Wang et al., 2021; Ding G et al., 2022). Specifically, porcine menisci were freshly harvested and then pulverized by cyclic freeze-thaw grinding and treated with DNase to obtain fine dmECM powders. Dried dmECM powders were then prepared to decellularized meniscus extracellular matrix (dmECM) by gradient centrifugation of pepsin-solubilized meniscus digest. Samples were stored at −20°C for further use.

A dynamic liquid system was utilized to fabricate three-dimensional aligned nanofiber yarn scaffold. PLLA-CL and dmECM (9:1, wt/wt) were dissolved in HFIP to yield to a 12% (wt/v) solution. The solution was fed at 1.2 mL/h and charged with a 12 kV voltage to generate nanofibers. Electrospun nanofibers were twisted and organized into yarns through a water vortex and finally collected on a mandrel (100 mm diameter, 60 rpm) to form nanofiber yarn scaffolds. The scaffolds were immediately frozen at −80°C and lyophilized for further use.

### 2.3 Scaffold characterization

The photographs of the native meniscus and nanofiber yarn scaffold were recorded by a digital camera. The morphology of samples was examined by a Phenom XL desktop scanning electron microscope (Phenom, Netherlands) at an accelerating voltage of 5 kV.

The diameter, angle distribution, and pore size of the native meniscus and scaffold were measured based on SEM images using Image J. 100 nanofibers were randomly selected from five different SEM images, and their diameters and angle distribution with respect to the longitudinal axis were measured (Xu et al., 2013). Pore size was determined from ∼25 pores randomly chosen in a typical SEM image (*n* = 5).

The porosity of native meniscus and scaffolds was determined by liquid displacement method as previously described (Wu et al., 2014). Cubic specimens were prepared from the native porcine meniscus and freeze-dried for testing. Specifically, the length (l), width (w) and height (h) of the scaffolds were measured with a vernier caliper. Dry samples were weighed (m_0_), immersed in ethanol for 1 h, and then weighed (m_1_) again (*n* = 6). The porosity of scaffold was calculated by the following equation: $porosity\;\left(\%\right)=\frac{100\times \left(m_{1}-m_{0}\right)}{\rho_{ethanol}\times l\times w\times h}$ .

Water absorption capacity of nanofiber yarn scaffold was calculated by equation: $Water\;absorption\;capacity\;\left(\%\right)=\frac{{m_{w}-m}_{d}}{m_{d}}\times 100\%$ . First, dry samples were weighed (m_d_) and then were transferred into deionized water and completely immersed for 1, 3, 5, 10, 20, 30, 60, 90, and 150 min. Wet samples were wiped on a paper and weighed (m_w_) (*n* = 3).

Degradation of the scaffolds was measured following our previous report with minor modifications (Li et al., 2021a). Scaffolds were cut into square pieces (∼10 mg) and weighed in dry state (w_0_). Then scaffolds were immersed in 2 mL phosphate-buffered saline (PBS), and incubated at 37°C, 100 rpm for up to 4 weeks. At each time point, samples were collected, washed five times with deionized water, lyophilized, and weighed (w_1_) (*n* = 3). Weight loss of scaffolds was calculated by the equation: $Weight\;loss\;\left(\%\right)=\frac{{w_{1}-w}_{0}}{w_{0}}\times 100\%$ .

Mechanical properties of native meniscus and electrospun yarn scaffold were determined in wet state. Specimens of native meniscus for tensile testing were harvested from the middle region of each lateral meniscus along the circumferential direction. Scaffolds were tailored into strips (10 × 40 mm) along the direction of fiber alignment and incubated in PBS at 37°C for 24 h. Samples were clamped by the grips of a universal testing machine (Instron 5567, Norwood, MA) with a 200 N load cell and stretched at a crosshead speed of 5 mm/min until failure. Ultimate tensile strength (UTS) was obtained from the point of maximum tensile strength, and Young’s modulus was calculated as the slope of the initial 5% linear portion from the stress-strain curve (*n* = 5).

### 2.4 Cell isolation and expansion

Isolation and culture of BMSCs: New Zealand white rabbits (2.5–3 kg) were used to aspirate bone marrow, and then cultured for 5 days without changing the medium to promote cell adhesion on the culture dish. The isolated BMSCs were cultured in high glucose Dulbecco’s Modified Eagle Medium (DMEM; Gibco, Grand Island, NY) supplemented with 10% fetal bovine serum (FBS; Hyclone, Logan, UT) and 1% penicillin/streptomycin for two passages before cell seeding (Wang X et al., 2022).

Human adipose-derived stem cells were extracted from adipose tissue by the Animal Protection and Experimental Committee of Shanghai Ninth People’s Hospital, Shanghai Jiao Tong University School of Medicine (SH9H-2018-T22-1). Briefly, adipose tissues were soaked in 0.25% chloramphenicol solution for 15 min, rinsed with PBS, and cut into small pieces. Tissues were digested with 2% collagenase IV at 37 °C for 1 h, and then centrifuged at 1,500 rpm for 5 min. ADSCs were cultured in high glucose DMEM supplemented with 10% FBS and 1% penicillin/streptomycin for two passages before cell seeding (Liu et al., 2008).

Articular chondrocytes were isolated from New Zealand White rabbits (2 weeks old). Articular cartilage tissues were harvested from the femoral condyle area and rinsed with PBS containing 0.25% chloramphenicol. Cartilage tissues were then digested in 0.2% collagenase type II for 8–10 h at 37°C and filtrated through a 70 μm cell strainer. Chondrocytes were expanded in high glucose DMEM supplemented with 10% FBS and 1% penicillin/streptomycin for two passages before cell seeding (Ding Y et al., 2022).

Scaffolds were punched into either 22- or 11-mm discs to fit 12-well or 48-well plates, respectively, disinfected with 70% ethanol, and washed thoroughly with PBS prior to cell seeding. Cells at 80% confluence were detached with trypsin, centrifuged, and resuspended in medium. The cell suspension (5.0 × 10^4^ cells/cm^2^) was seeded onto the surface of the scaffolds and incubated in regular culture medium at 37°C, 5% CO_2_. Samples were harvested at 1, 14, and 28 days for further analyses.

### 2.5 Proliferation, viability, and morphology of cells

The proliferation rate of cells cultured on scaffolds was determined by Cell Counting Kit-8 (CCK-8, Beyotime Biotechnology, Shanghai, China) assay following the manufacturer’s instructions. Cell-seeded scaffolds were incubated with CCK-8 for 2 h at 37°C, and then the optical density (OD) was read at 450 nm using a plate reader (Multiskan MK3, Thermo, United States) (*n* = 3).

Cell viability was evaluated by live/dead staining. Scaffolds were briefly rinsed with PBS, stained with a Live and Dead Cell Viability Assay (Invitrogen, United States) for 30 min at 37°C to visualize live and dead cells, and imaged with a confocal microscope (Nikon, Japan).

To visualize the morphology of cells cultured on scaffolds, cell-seeded scaffolds were fixed in 4% paraformaldehyde, dehydrated with gradient ethanol, and freeze-dried. Samples were then sputter-coated with gold and imaged using a scanning electron microscope.

### 2.6 Biochemical analysis

The cell-seeded scaffolds (*n* = 3) were collected to perform meniscus-related biochemical evaluations for cellular double-stranded DNA (dsDNA), sulfated glycosaminoglycan (sGAG), total protein content and total collagen content by using PicoGreen dsDNA assay (Invitrogen, United States), dimethylmethylene blue assay (Sigma-Aldrich), BCA assay and hydroxyproline assay (Jiancheng Bioengineering Institute, Nanjing, China).

Samples were digested by papain at 65°C for 24 h and then centrifuged at 2,980 g for 10 min. The supernatant was extracted and centrifuged at 10,000 g in phenol/chloroform/isopentyl alcohol (25:24:1) for 30 min. DNA was extracted from the aqueous layer with 3 M sodium acetate/ethanol solution (v/v = 1:20) at −20°C overnight. DNA content was determined using the PicoGreen dsDNA assay in accordance with the manufacturer’s instructions (*n* = 3).

The GAG content was analyzed by spectrophotometric microreader with dimethylmethylene blue as previously described (Yan et al., 2009). Briefly, GAG was precipitated by guanidinium chloride solution (0.98 mol/L). OD value was determined at 595 nm after dissolving the GAG precipitate. The concentration of GAG was calculated against a calibration curve with known concentrations of chondroitin-4-sulfate (*n* = 3).

The total protein produced by cells on scaffolds was determined by a BCA assay. Cell-seeded scaffolds were lysed by cell lysis buffer and centrifuged at 13,000 rpm for 5 min at 4°C. The supernatant of centrifuged lysates was incubated with BCA solution in a 37°C incubator for 30 min. The absorbance at 562 nm of BCA incubation was read using a Multiskan MK3 plate reader (*n* = 3). The amounts of total protein were calculated against a standard curve of known concentrations of bovine serum albumin.

The total collagen content was determined by a hydroxyproline assay. Samples were hydrolyzed using 6 M hydrochloric acid and incubated with equal volume of chloramine T solution for 20 min followed by reacting with color reagent at 65°C for 20 min. The absorbance of solution at 558 nm was read using a plate reader. The concentration of hydroxyproline was calculated against a calibration curve with known concentrations of hydroxyproline (*n* = 3). Collagen content was calculated from hydroxyproline using a conversion factor of 14.3% (Sawkins et al., 2013).

### 2.7 Real-time polymerase chain reaction (RT-PCR) analysis

Expression of meniscus-associated genes (COL I, COL II, SOX 9, ACAN) was analyzed by RT-PCR. Total RNA was extracted from cell-seeded scaffolds (*n* = 3) using Trizol (Sangon Biotech Co.,Ltd, Shanghai, China) at day 28. cDNA was synthesized using RevertAid First Strand cDNA Synthesis kit (Thermo Fisher Scientific) according to the manufacturer’s instructions. RT-PCR was performed using NovoStart^®^ SYBR qPCR SuperMix Plus (Novoprotein Scientific Inc., Shanghai, China) in an Applied Biosystems™ 7500 real-time PCR system and analyzed by comparative Ct quantification method (∆∆Ct). The sequences of primers used for meniscus-associated genes are shown in Supplementary Table S1. The expression levels of genes were relative to glyceraldehyde 3-phosphate dehydrogenase (GADPH) and normalized to the level of collagen II of each type of cell.

### 2.8 Histological and immunohistochemical analyses

Cell-seeded scaffolds were fixed in 4% paraformaldehyde, embedded in paraffin, and cut into 5 μm thick sections. The slices were stained with hematoxylin and eosin (H&E) and Alcian blue staining respectively following manufacturer’s instructions. For immunohistochemical (IHC) staining, sections were incubated with primary antibodies [rabbit monoclonal anti-collagen I (Abcam) and rabbit monoclonal anti-collagen II (Abcam)] overnight at 4°C. Samples were then incubated with peroxidase-conjugated goat-anti-mouse secondary antibody (Jackson ImmunoResearch Laboratories, PA) and developed with diaminobenzene (DAB) kit (Sigma) for visualization. The histological and immunohistochemical staining samples were visualized using a fluorescence scanner (panoramic MIDI, Hungary).

### 2.9 Biomechanical properties of cell-seeded scaffolds

Mechanical properties of cell-seeded scaffolds (22-mm diameter) were determined 14, and 28 days after cell seeding. Cell-seeded scaffolds were tailored into strips of 5 mm × 20 mm along the direction of fiber alignment and tested as described above (*n* = 6). Cell-free scaffolds incubated under the same conditions served as controls (*n* = 6).

### 2.10 Statistical analysis

Data are presented as mean ± standard deviation. Statistical analysis was analyzed by one-way analysis of variance (ANOVA) with Tukey’s *post hoc* multiple comparisons. A *p*-value less than 0.05 was considered statistically significant.

## 3 Results

### 3.1 Scaffold properties

**FIGURE 1:**
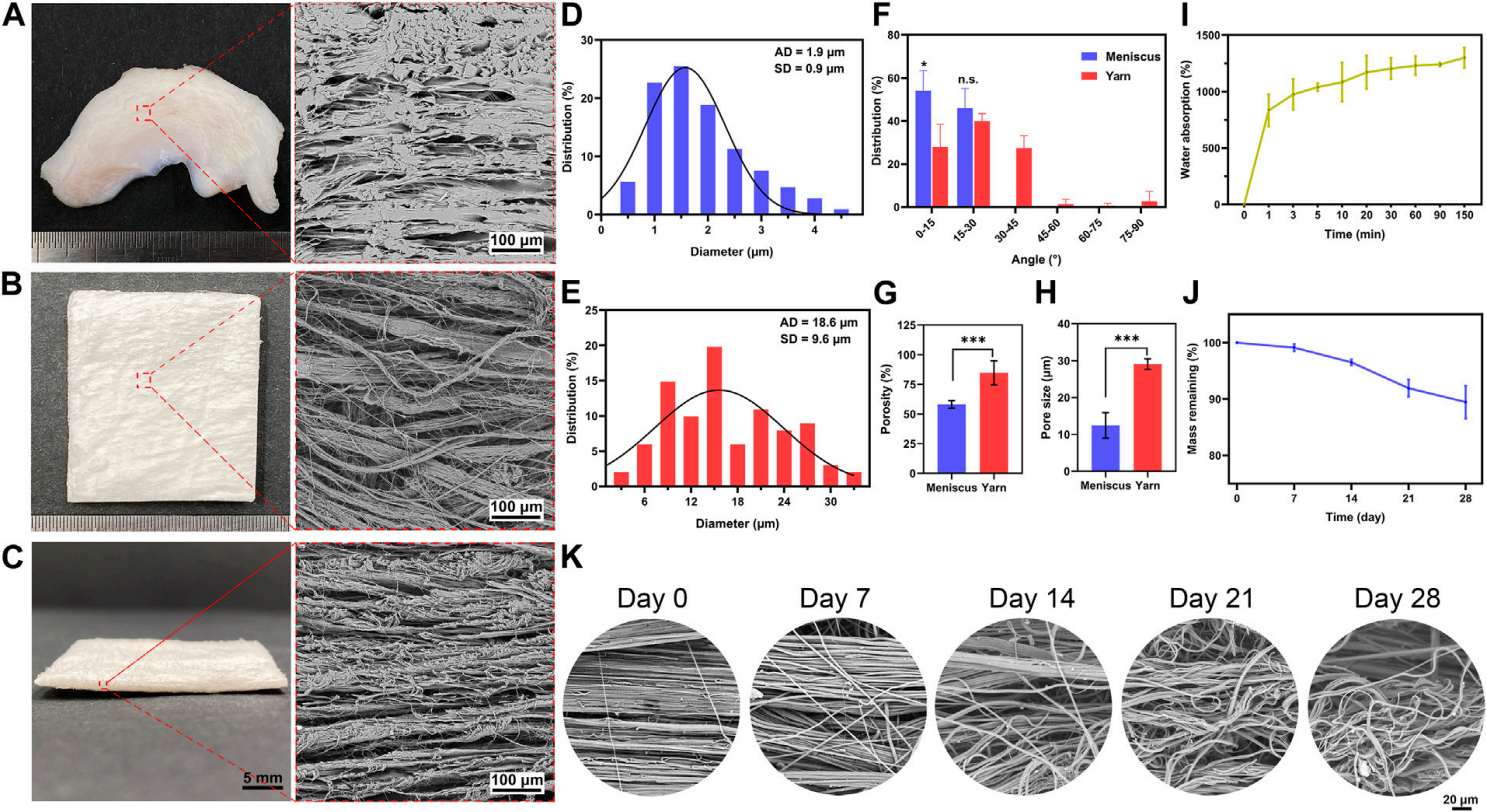
Physicochemical properties of electrospun nanofiber yarn scaffold. The native meniscus shows dense ultrastructure of hierarchical ECM **(A)** with an average diameter of approximately 2 µm **(D)** and a fiber angle distribution of 0–30° **(F)**. SEM images **(B, C)** reveal that the scaffold exhibits aligned fiber yarns with an average diameter of approximately 20 µm **(E)** and fiber angle distribution of 0–30° **(F)**, which resemble the hierarchical ECM of the native meniscus. The scaffold has a higher porosity **(G)** and pore size **(H)** than the native meniscus. The scaffold shows 1,300% water absorption **(I)**. Despite a 10% weight loss after 28 days of *in vitro* incubation **(J)**, it shows significant morphology change that aligned fibers become swollen fibers with coils **(K)**. * *p* < 0.05, *** *p* < 0.001.

The morphology of the native meniscus in cross-section and nanofiber yarn scaffold is observed by SEM. The native meniscus showed dense and hierarchical bundles of collagen fibers oriented along the curved lunar meniscus (Figure 1A), with an average fiber diameter of approximately 2 μm. The scaffold showed aligned fiber yarns with an average diameter of approximately 20 μm (Figure 1E) and exhibited a loose and porous structure between the yarns (Figures 1B, C), showing a structure similar to that of the native meniscus (Figure 1A). The scaffold had a porosity of 85% ± 10% (Figure 1G) and groove width of 30 ± 1 μm (Figure 1H), which showed a porous structure with large pores. The scaffold exhibited a larger porosity and pore size than the native meniscus (Figures 1G, H), thus providing good conditions for cell growth. The scaffold showed a UTS of 2 ± 0.3 MPa, Young’s modulus of 5.6 ± 0.8 MPa, and breaking strain of 99% ± 10%, while the native meniscus showed a UTS of 5.3 ± 0.3 MPa, Young’s modulus of 15.6 ± 4.2 MPa, and breaking strain of 36% ± 9% (Supplementary Table S2). The distribution of fiber angle was evaluated to determine the fiber alignment of the scaffold (Figure 1F). The result showed that the fiber angles of scaffold were mainly concentrated between 0 and 30° (0° is defined as the fiber orientation parallel to the horizontal axis) (Figure 1F), indicating that the scaffold had a high degree of orientation, mostly aligned in the parallel direction, which is similar to the native meniscus structure. The scaffold showed a water absorption rate of approximately 1300%, which reached about 1000% in 3 min (Figure 1I). The scaffold showed a slow *in vitro* degradation rate during the first 14 days followed by a slightly accelerated degradation rate thereafter, leading to a residual mass of 90% ± 3% at day 28 (Figure 1J). Figure 1K demonstrates the morphology change during the degradation of the scaffold, where the oriented aligned fibers become swollen fibers with coils (Figure 1K).

### 3.2 Cell proliferation and morphology

**FIGURE 2:**
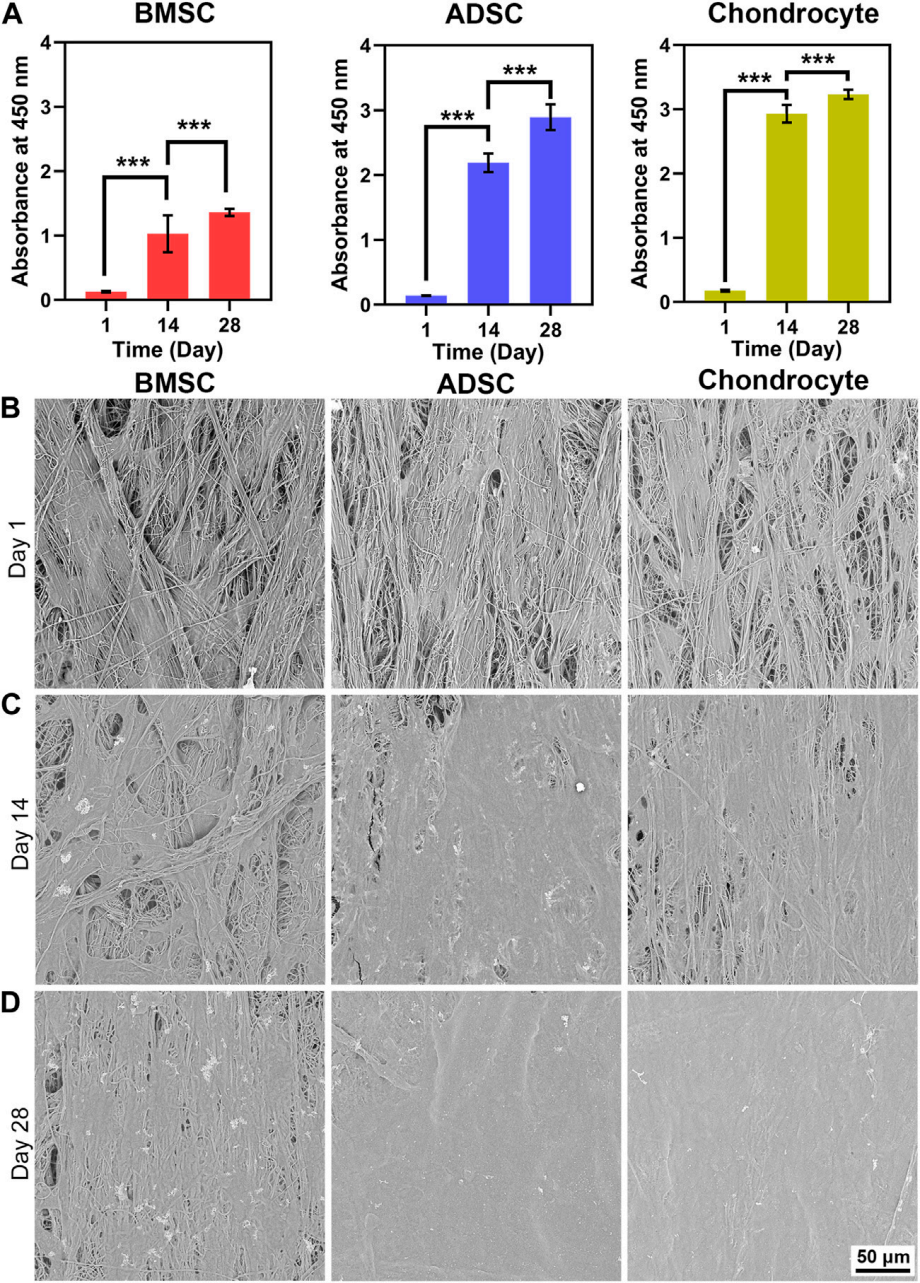
Cell proliferation and morphology on electrospun yarn scaffolds. CCK-8 assay reveals that cells proliferate robustly **(A)**, which is confirmed by increased cell coverage on scaffolds from day 1–28 **(B–D)**. *** *p* < 0.001.

**FIGURE 3:**
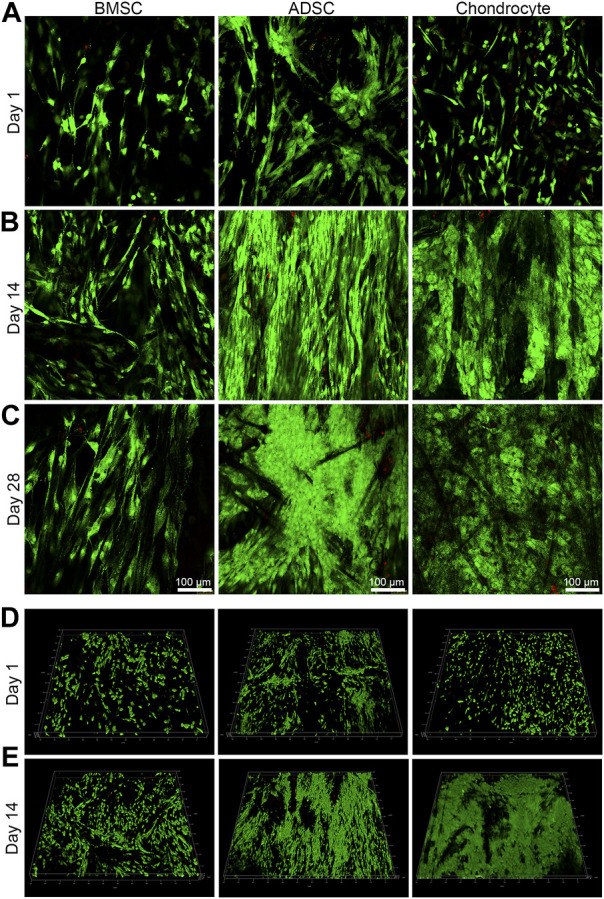
Cell viability and distribution on scaffolds. Live/dead staining demonstrates that cells are predominantly live with a spindle shape at day 1 **(A)**. BMSC and ADSC show increased numbers and maintain spindle shape and grow along fiber direction, while chondrocyte undergoes significant morphology change into round shape from day 14 up to day 28 **(B, C)**. Reconstructed images illustrate that cells experience three-dimensional growth pattern on electrospun yarn scaffolds **(D, E)**.

The proliferation, viability, and morphology of cells cultured on scaffolds were assessed by CCK-8 assay, SEM, and live/dead staining (Figure 2, 3), respectively. CCK-8 assay demonstrates that proliferation rates of BMSC, ADSC, and chondrocyte greatly increased after 14 days. After that, cells maintained active growth for up to 28 days but the proliferation rate slowed down. BMSC showed much smaller increase in cell number in comparison with ADSC and chondrocyte (Figure 2A).

SEM images reveal the morphology and distribution of cells on the scaffold. BMSC, ADSC, and chondrocyte firmly adhered to scaffold fibers and were evenly distributed along the fiber direction at day 1 (Figure 2B). At day 14, increased cell coverage on the scaffold surface was observed at day 14 (Figure 2C). At day 28, ADSC and chondrocyte formed a confluent layer and completely covered scaffold surface (Figure 2D). Moreover, BMSC coverage on scaffolds were lower than ADSC and chondrocyte at days 14 and 28, which is in line with the semi-quantitative results that the number of BMSC was significantly lower than that of ADSC and chondrocyte.

Live/dead staining also indicate good cytocompatibility of the scaffold (Figure 3). BMSC, ADSC and chondrocyte showed a predominance of viable cells (green) with a small number of dead cells (red). At day 1, individual cells could be observed on the surface of the scaffold, and all three cells were spindle-shaped and elongated in the direction of the fibers (Figure 3A). At days 14 and 28, numbers of ADSC and chondrocytes greatly increased, while BMSC also showed an evident increase in cell population but not as much as ADSC or chondrocyte. In addition, BMSCs underwent evident hypertrophic change by increased cell area from day 14 to day 28. Fluorescent images reveal that ADSC and chondrocyte grow in the direction of fiber bundles, forming a hierarchically oriented structure similar to the natural meniscus (Figures 3B, C). Three-dimensional confocal images at days 1 and 14 showed that three cells exhibited show good infiltrative growth on the scaffold (Figures 3D, E). At the same time, a significant transformation of chondrocyte morphology can be observed, whereas neither BMSC nor ADSC did (Figures 3A–C). At day 1, chondrocytes showed a predominant spindle-shaped morphology (Figure 3A), and a switch of spindle-shaped cells to spherical cells can be observed at day 14 and day 28, forming larger size chondrocytes cluster on the fiber bundle (Figures 3B, C).

### 3.3 DNA content, biochemical analysis, and expression of meniscus related genes

**FIGURE 4:**
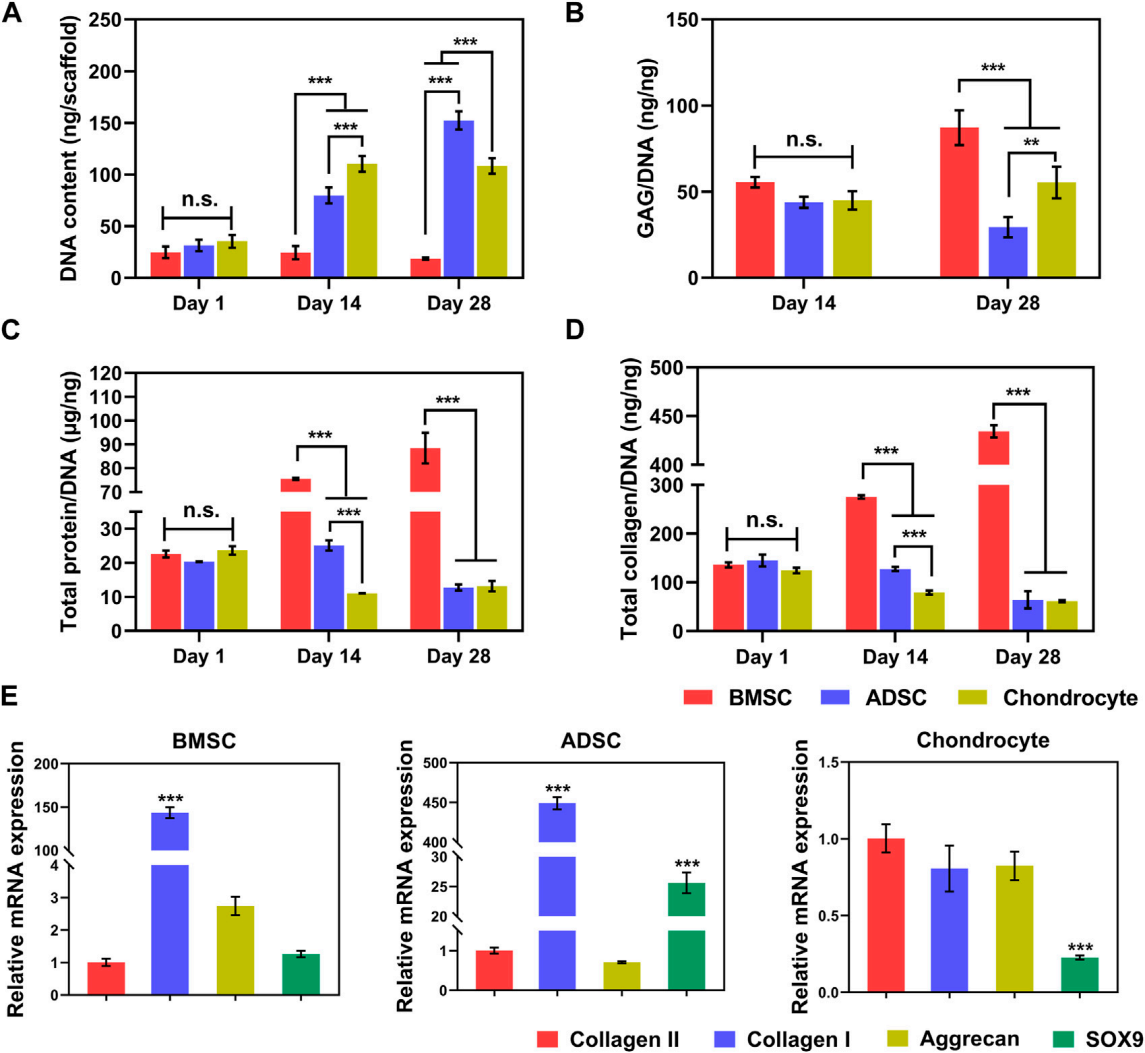
Biochemistry and gene expression of cells on scaffolds. Increased total DNA contents indicate steady cell proliferation **(A)**. At per cell level, BMSC produces significantly greater GAG **(B)**, total protein **(C)**, and collagen **(D)** than ADSC and chondrocyte. BMSC and ADSC show predominate collagen I expression, in contrast, chondrocyte maintains high level of collagen II expression **(E)**. * *p* < 0.05, ** *p* < 0.01, *** *p* < 0.001.

Quantification of total DNA content shows that the DNA contents of ADSC and chondrocyte increased significantly with the culture time (*p* < 0.001). In contrast, the total DNA content of BMSC was unchanged (*p* > 0.05) for up to 28 days (Figure 4A), which is consistent with the CCK-8 quantification.

Biochemical components of GAGs, total protein, and collagen produced by cells on scaffolds increased along with prolonged time (Figures 4B–D). Interestingly, despite the poor proliferation of BMSC on the scaffold, BMSC had greater production of GAG (Figure 4B) at day 28, total protein (Figure 4C), and total collagen (Figure 4D) at days 14 and 28 per cell (*p* < 0.001). In addition, chondrocytes secreted significantly higher GAG at per cell level than those of ADSC at day 28. At the cellular level, BMSCs are likely to produce the major components of meniscal matrix, while chondrocytes are more active in proliferation as evidenced by much greater total DNA (Figure 4A) and thus the chondrocyte population produced greater total amounts of ECM products on the yarn scaffold than BMSCs (data not shown).

Meniscus-related gene expression of cells on the scaffold was determined by real-time PCR (Figure 4E). BMSC and ADSC predominately expressed collagen I, while the cartilage-related genes collagen II and aggrecan were much lower or even minimal (*p* < 0.001). Chondrocytes expressed comparable gene levels of collagen I, collagen II, and aggrecan with a significantly lower level of SOX9, indicating that chondrocytes maintained a major chondrogenic phenotype with a trend in fibroblastic differentiation.

### 3.4 Histological and IHC analyses

**FIGURE 5:**
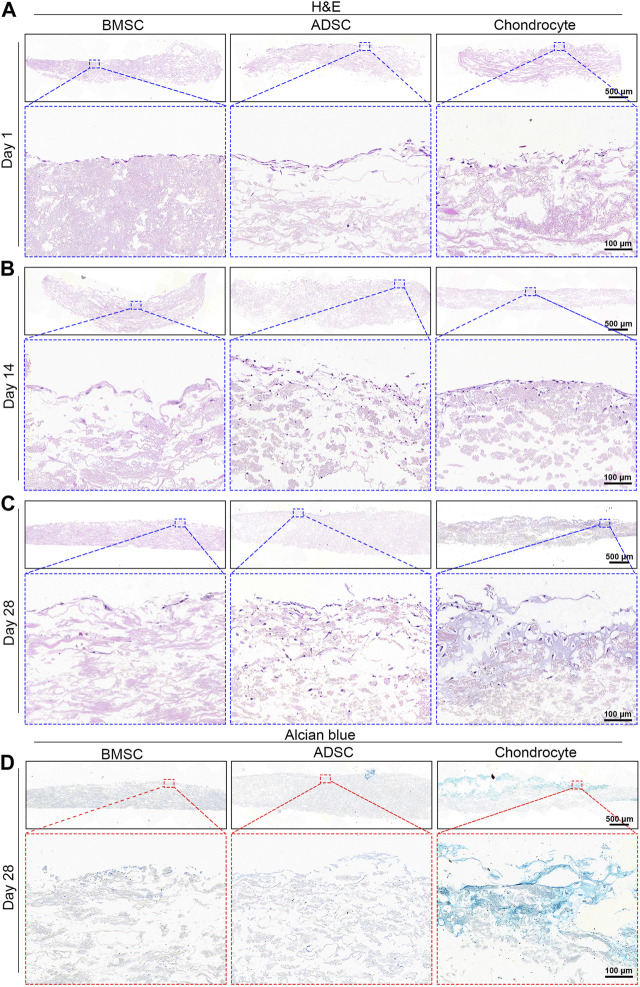
Histological analysis of cell-seeded scaffolds. H&E staining shows increased cell infiltration into scaffold over time **(A–C)**. Chondrocytes show intense positive Alcian blue staining while BMSC and ADSC are absent of Alcian blue staining **(D)**.

Infiltration and morphology of cells were visualized by H&E and Alcian blue staining (Figure 5). H&E staining demonstrates evident morphological change in the nucleus and cytoplasmic of cells (Figures 5A–C). From day 1 to day 28, cells showed a tendency to grow downward along the sparse and porous nanofiber yarns. In comparison to BMSC, ADSC and chondrocyte had greater cell density as well as deeper infiltration depths with abundant matrix filling the pores of the scaffold at days 14 and 28. Notably, chondrocytes showed morphological change within the scaffold over time. Especially at day 28, a significant increase in cartilage matrix was observed, with typical cartilage cavitation and clear chondrocyte lacunae (Figure 5C). Alcian blue staining is used to determine sulfated proteoglycan distribution within the cell-seeded scaffolds after 28 days. Chondrocytes produced and were embedded a large amount of GAG-rich extracellular matrix, confirming the formation of cartilage lacuna structure (Figure 5D). BMSC and ADSC were largely in the absence of GAG-rich extracellular matrix.

**FIGURE 6:**
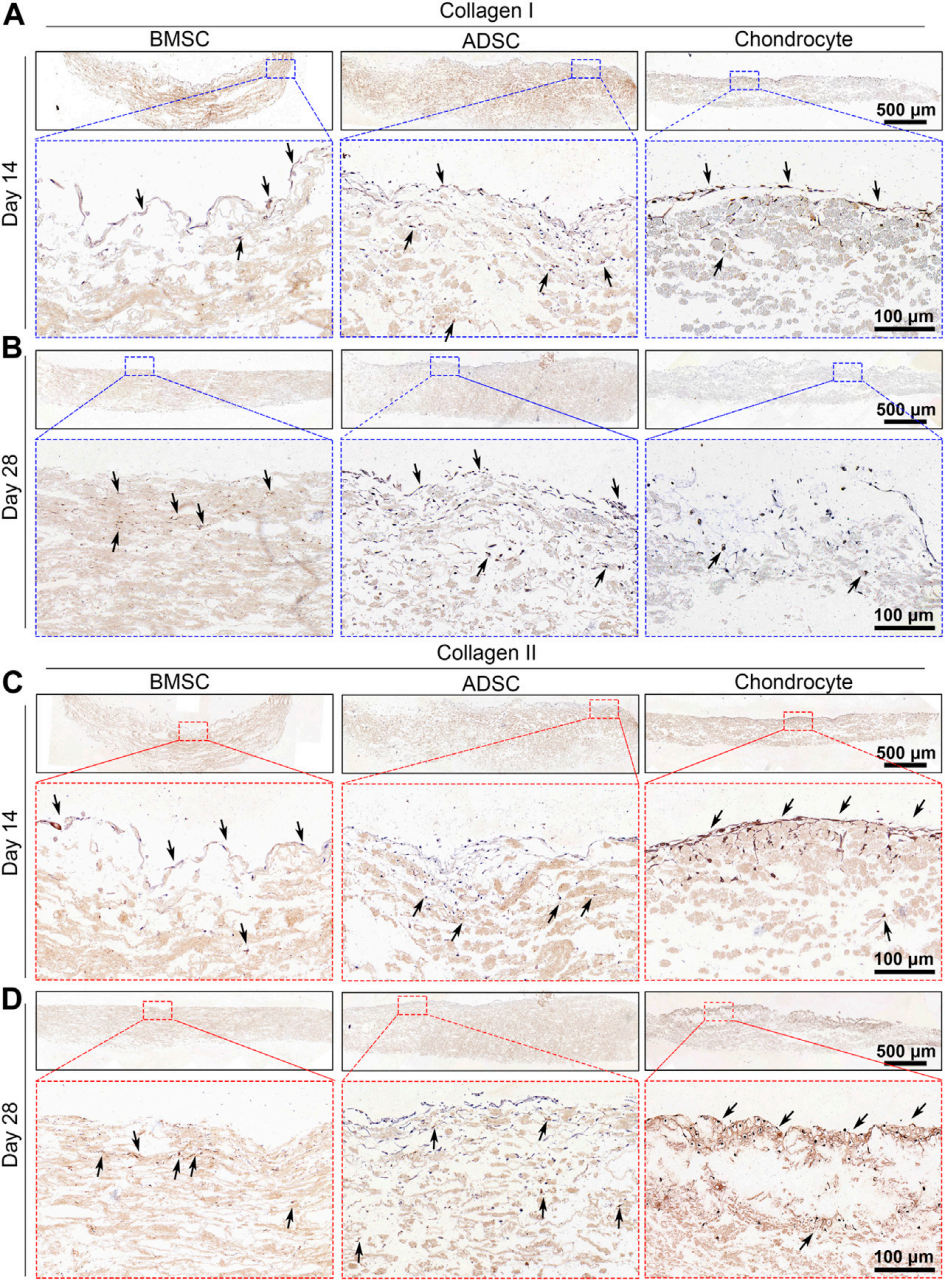
Immunohistochemical staining of cell-seeded scaffolds. BMSC and ADSC are positive in collagen I staining **(A, B)** but are negative in collagen II staining **(C, D)**. A small portion of chondrocyte shows positive collagen I staining and most chondrocytes are positive in collagen II staining.

IHC analysis was performed to further validate the phenotype of cells on the scaffold. BMSC and ADSC were positive for collagen I staining, while only a small portion of chondrocytes were positive for collagen I expression at days 14 and 28 (Figures 6A, B). BMSC and ADSC were negative for collagen II staining, in contrast, chondrocytes showed intensive positive collagen II staining (Figures 6C, D).

### 3.5 Mechanical properties of cell-seeded constructs

**FIGURE 7:**
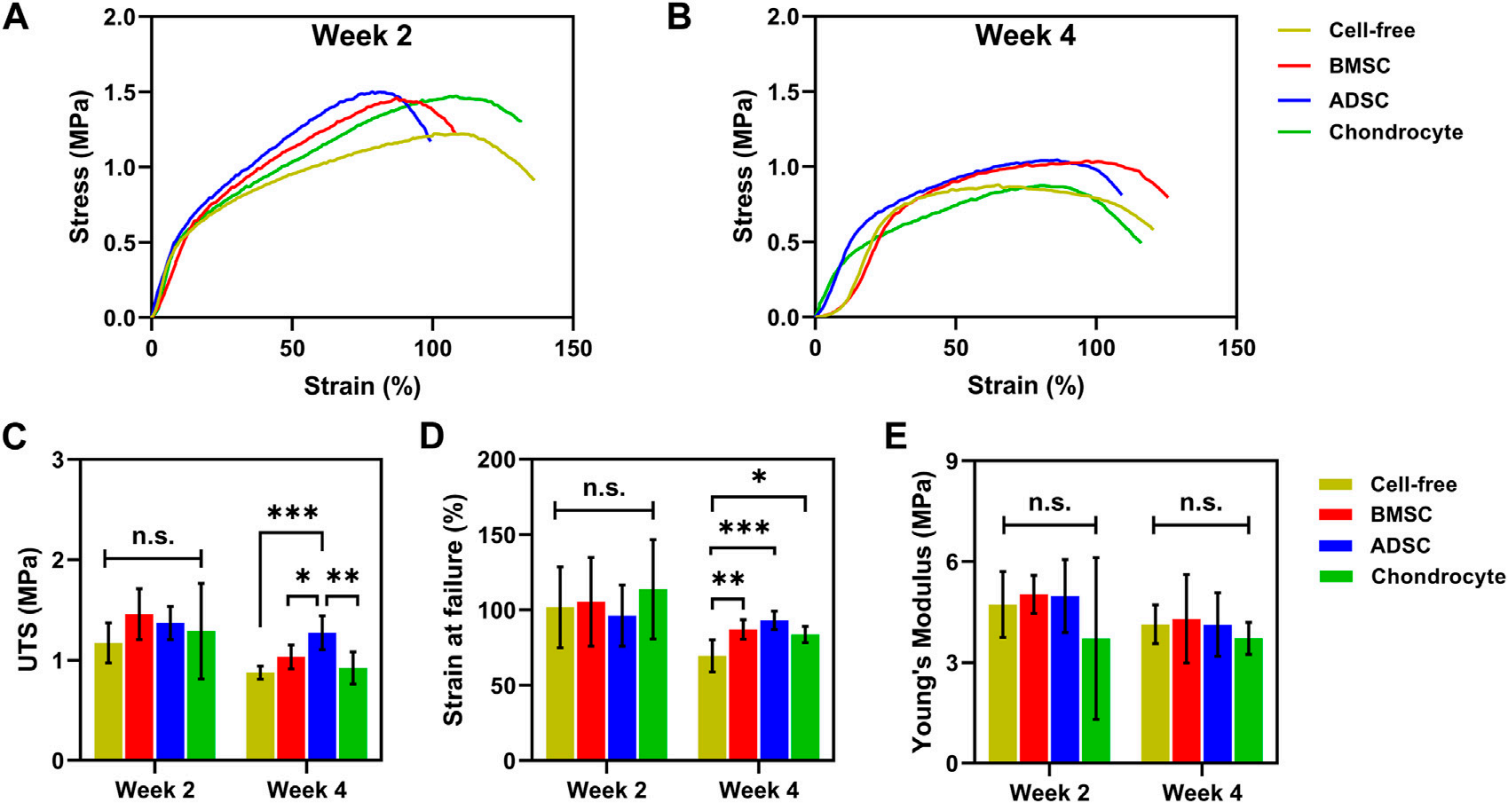
Biomechanics of cell-seeded scaffolds. Cell-seeded scaffolds show similar typical stress-strain curves to cell-free scaffolds **(A)**. BMSC and ADSC-seeded scaffolds exhibit greater UTS **(C)** and breaking strains **(D)** than cell-free scaffolds at day 28. No significant change in Young’s modulus is observed between cell-seeded and cell-free scaffolds **(E)**. * *p* < 0.05, ** *p* < 0.01, *** *p* < 0.001.

A uniaxial tensile test was conducted to determine biomechanics of cell-seeded scaffolds. The stress-strain curves showed an overall decreasing trend over time, which is associated with scaffold degradation. Stem cell-seeded scaffolds had higher curves than the cell-free scaffold after 28 days, indicating reinforcement effect of stem cell-secreted ECM products on scaffolds (Figures 7A, B). No significance in mechanical properties was observed among those cell-seeded and cell-free scaffolds at day 14. Scaffolds seeded with BMSC (1 ± 0.1 MPa) and ADSC (1.3 ± 0.2 MPa) had significantly higher UTS than the cell-free scaffold (0.9 ± 0.1 MPa) and chondrocyte (0.9 ± 0.2 MPa) (Figure 7C) at day 28. Meanwhile, cell-seeded scaffolds showed significantly increased breaking strains at day 28 (*p* < 0.05) (Figure 7D). Cells did not show evident effect on the moduli of scaffolds for up to 28 days (Figure 7E). These results show that stem cells could compensate for the tensile loss of the scaffold to some extent, while the compensation of chondrocyte was not significant, probably due to the low protein production.

## 4 Discussion

Many cell sources have been proposed for meniscus repair and regeneration due to the complexity of cellular biology of the meniscus, while the optimal cell source remains unassured. The objective of this study is to assess the feasibility and efficacy of BMSC, ADSC, and articular chondrocytes for constructing engineered meniscus with biomimetic scaffolds toward an overreaching goal to examine their potential for meniscus repair. Our results show that electrospun nanofiber yarn scaffolds are beneficial to these cells for their proliferation and extracellular matrix production as well as the maturation of engineered tissue constructs. Stem cells and chondrocytes underwent differential growth and phenotypical changes and generated distinct patterns of extracellular matrix accumulation, which lead to various biomechanical properties of cell-seeded constructs. This study is significant because it provides evidence to show that chondrocytes are superior to maintaining chondrogenesis within the yarn scaffold and repairing the inner part of the meniscus, while the BMSCs and ADSCs are likely to form fibrocartilaginous tissue within the yarn scaffold and might be suitable for repair the outer part of the meniscus.

Robust cell proliferation is essential for repairing and regenerating defective tissues in cell-based regenerative approaches. Previously, we have proven that electrospun nanofiber yarn scaffolds provide a good microenvironment for cells and boost cell proliferation and assembly into three-dimensional cell-matrix constructs (Wu et al., 2012; Wu et al., 2014; Li et al., 2021b; Li et al., 2021c; Wang et al., 2021). Recently, we and other groups demonstrate that incorporating dmECM into electrospun nanofibers might lead to conducive scaffolds or meniscus repair (Gao et al., 2017; Li et al., 2021b; Wang et al., 2021; Xia et al., 2021; Dorthe et al., 2022; Stocco et al., 2022). Our recent studies show that dmECM contains multiple meniscus-tissue specific bioactivities including collagen and GAGs that are essential for cell proliferation (Ding G et al., 2022). In line with these studies, we show that ADSCs and chondrocytes proliferate robustly within yarn scaffolds (Figures 2, 3). In contrast, BMSCs show poor proliferation, which might be associated with the absence of growth factor stimulation. It should be noted that BMSCs showed the greatest ECM production at the per cell level. It is likely that a cell produces less ECM when experiencing a higher proliferation rate at per cell level, that is, an actively growing cell is not active in producing ECM products. It is well known that the proliferation of BMSCs is largely growth factor dependent. Our previous study has demonstrated that TGF-β1 significantly boosts the proliferation of BMSCs within electrospun yarn-based scaffolds in comparison to the TGF-β1-free control (Zheng et al., 2016). In this study, we did not supplement growth factor into the culture medium for a head-to-head comparison of various cells and their response to electrospun yarn scaffolds. Our results provide important implications for various cell sources in meniscus repair: growth factor stimulus is essential for BMSC-based approach, while growth factor boost seems dispensable for ADSC and chondrocyte.

Nanofiber configuration of electrospun scaffolds dictates cell-matrix interactions. Electrospun nanofiber yarn scaffolds are featured by their aligned bundles similar to the circumferential collagen fibrils. This unique biomimetic fibrous structure guides cell elongation along the fiber direction and leads to spindle-shaped cells at day 1 (Figure 3). Recent studies have shown that chondrocytes exhibited consistent circular, flatten morphology on conventional electrospun randomly oriented nanofiber scaffolds (Gao et al., 2017; Xia et al., 2021). However, in this study, chondrocytes recognized and elongated along the aligned fiber bundle structure initially and then underwent an evident morphological transition from elongated to circular shape with reduced cell spreading on electrospun yarn scaffolds. The unique fiber bundle structure of electrospun yarn scaffolds is probably the key mediator for the morphological change of chondrocytes. Chondrocytes are shaped by the three-dimensional topographic nanofiber bundles to elongated spindles shortly after cell seeding. In a previous report demonstrated that dmECM provides key bioactive for chondrocytes to maintain circular morphology, even on electrospun aligned nanofiber membranes (Gao et al., 2017). Therefore, later morphological transition is likely attributed to the presence of dmECM. Interestingly, after 28 days, chondrocytes secreted abundant GAG-rich extracellular matrix that was embedded in yarn scaffolds and formed typical chondrocyte lacunae alike the native cartilage tissue. These results indicate that electrospun yarn scaffolds provide conducive microenvironments for chondrocytes to maintain chondrogenic phenotype, which might potentiate the repair and regeneration of the white zone of the meniscus.

Fibroblast-like cells and chondrocytes represent the dichotomy of resident cells in native meniscus. BMSCs showed gradually hypertrophic change over time, while ADSCs maintained it typical spindle shape on electrospun yarn scaffolds (Figure 3). It is reported that reduced cell area and morphological change to circular phenotype of stem cells are indicative of mesenchymal chondrogenesis (McCorry et al., 2016). Together with strong collagen I gene expression, both BMSC and ADSC are tended to undergo fibroblastic differentiation on electrospun yarn scaffolds. In our recent studies, we reported that meniscus cells maintained an elongated morphology with hypertrophic trend on electrospun yarn scaffolds (Li et al., 2021c), which is similar to that of fibroblasts (Li et al., 2021a) and BMSCs and ADSCs on electrospun yarn scaffolds. Taken together, these findings indicate that stem cells are likely to undergo fibroblast differentiation on electrospun yarn scaffolds. McCorry et al. (2016) reported that BMSCs transitioned from spindle morphology to circular shape with reduced hypertrophy when cocultured with meniscus cells. Future studies shall investigate the coculture of stem cells with chondrocytes for constructing fibrocartilage tissue and meniscus repair. For cell-scaffold-based approaches to repair the meniscus, it is critical to select appropriate cell sources. Our findings demonstrate that stem cells and chondrocytes might be separately seeded on the outer and inner portions of electrospun yarn scaffolds, respectively, to construct regionally specific tissue-engineered meniscus.

Cell-material interactions dominate the biochemical and biomechanical properties of cell-seeded constructs. Both stem cells and chondrocytes generate extracellular matrix products on electrospun yarn scaffolds. Collagen is the major extracellular matrix component contributing to tensile strength, while GAG accounts for the elastic properties of tissues. BMSC and ADSC deposited significantly higher amounts of collagen than chondrocytes, which gives rise to the increased tensile strength of stem cell-seeded constructs (Figure 7). Our previous studies also illustrate similar reinforcement effects of fibroblasts and meniscus cells on the tensile strength of cell-seeded constructs. In contrast, chondrocytes deposited collagen but showed negligible contribution to the tensile strength. On the other hand, chondrocytes produced greater amounts of GAG on yarn scaffolds, unfortunately, it is difficult to measure the compression strength of chondrocyte-seeded scaffolds due to their limited thickness.

There are some limitations in this study. Although cells show gradual infiltration into the inner part of electrospun yarn scaffolds, the top side has significantly greater populations of cells than the bottom. Consequently, chondrocytes mature and form cartilage-like tissue on the top side. Future studies should focus on better experimental design and characterization of scaffolds (Lee et al., 2014) and apply dynamic culture systems and increase cell seeding density on scaffolds to induce full-thickness cell infiltration. In addition, we have well-established protocols for the isolation, identification, and expansion of primary stem cells, BSMC and ADSC, used in this study were not characterized or sorted and therefore are likely heterogeneous populations.

## 5 Conclusion

This study shows that electrospun yarn scaffolds are suitable to combine with stem cells and chondrocytes for constructing tissue-engineered meniscus. Chondrocytes maintained a stable chondrogenic phenotype and form cartilage-like tissue, which might be used to repair the white zone of the meniscus. Stem cells, especially ADSCs, are more likely to form fibroblastic tissue with yarn scaffolds in the absence of growth factor. Future studies shall investigate the coculture of stem cells and chondrocytes on yarn scaffolds for meniscus repair.

## Data Availability

The original contributions presented in the study are included in the article/Supplementary Material, further inquiries can be directed to the corresponding authors.
