# Supplementary material for: A comparative study on various cell sources for constructing tissue-engineered meniscus
Source: Front Bioeng Biotechnol. 2023 Mar 16;11:1128762. doi: 10.3389/fbioe.2023.1128762 (PMC10061001; doi:10.3389/fbioe.2023.1128762)
Supplement: Supplementary file 1 [file Table1.docx]

**Supplemental Materials**

**Table S1.** Primer sequence for RT-PCR analysis.

| Gene | Sequence (5’ - 3’) |
| --- | --- |
| rabbit β-actin | Forward: GCAGAA ACGAGACGAGATTG  Reverse: GCAGAACTTTGGGGACTTTG |
| rabbit collagen I | Forward: AAGAGCCTGAGCCAGCAGAT  Reverse: AGCCTTGGTTGGGGTCAAT |
| rabbit collagen II | Forward: TCCTGTGCGACGACATAATCT  Reverse: GCAGTGGCGAGGTCAGTAG |
| rabbit aggrecan | Forward: CACCCCGAGAATCAAATGGA  Reverse: TGGGCAGCGAGACCTTGT |
| rabbit SOX9 | Forward: AGGTGCTCAAGGGCTACGAC  Reverse: TTGACGTGGGGCTTGTTCT |
| human β-actin | Forward: AAGGTGACAGCAGTCGGTT  Reverse: TGTGTGGACTTGGGAGAGG |
| human collagen I | Forward: AGGGCCAAGACGAAGACATC  Reverse: GTCGGTGGGTGACTCTGAGC |
| human collagen II | Forward: CAGGATGGGCAGAGGTAT  Reverse: CGTCTTCACAGATTATGTCGT |
| human SOX9 | Forward: GATGAAATCTGTTCTGGGAATGT  Reverse: AACTGCTGGTGTTCTGAGAGG |
| human aggrecan | Forward: GCCAGCACCACCAATGTAAG  Reverse: CCTCCACGAACTCAGAAGTGAT |

**Table S2.** Physicochemical properties of the native meniscus and electrospun yarn scaffold.

|  | Pore size | UTS | Young’s modulus | Strain at failure | |
| --- | --- | --- | --- | --- | --- |
| Native meniscus | 12 ± 3.4 μm | 5.3 ± 2 MPa | 15.6 ± 4.2 MPa | | 36 ± 9% |
| Yarn scaffold | 29 ± 1.5 μm | 2 ± 0.3 MPa | 5.6 ± 0.8 MPa | | 99 ± 10% |
